# Spontaneous Tumor Lysis Syndrome in Small-Cell Lung Cancer: A Rare Complication

**DOI:** 10.14740/wjon946w

**Published:** 2015-10-26

**Authors:** Chanudi Weerasinghe, Mazen Zaarour, Sami Arnaout, Gwenalyn Garcia, Meekoo Dhar

**Affiliations:** a Department of Medicine, Staten Island University Hospital, North Shore - LIJ Health Care System, Staten Island, New York, USA; b Department of Medicine, Division of Hematology/Oncology, Staten Island University Hospital, North Shore - LIJ Health Care System, Staten Island, New York, USA

**Keywords:** Tumor lysis syndrome, Spontaneous tumor lysis syndrome, Small-cell lung cancer, Lung cancer

## Abstract

Tumor lysis syndrome (TLS) is a life-threatening condition which consists of a constellation of electrolyte imbalances, acute renal failure, seizure, and arrhythmias. It is most commonly seen with hematologic malignancies after the initiation of chemotherapy. However, it can also occur spontaneously, prior to treatment with cytotoxic agents. TLS has been rarely described with non-hematologic solid tumors, and it is even more uncommon to have spontaneous tumor lysis syndrome (STLS) in solid tumors. To our knowledge, only two cases of STLS in small-cell lung cancer (SCLC) were reported in the literature. Herein, we present the case of a patient with metastatic SCLC who developed STLS. Our case highlights that in the setting of metastatic solid tumors, STLS must be in the differential diagnosis, to allow prompt initiation of prophylaxis and treatment.

## Introduction

Tumor lysis syndrome (TLS) is an oncologic emergency caused by the rapid necrosis of tumor cells, leading to hyperphosphatemia, hyperkalemia, hyperuricemia and hypocalcemia [1, 2]. Consequently, it can lead to acute renal failure, heart failure, cardiac dysrhythmias, seizure and death. Therefore, this syndrome should be recognized in a timely manner, and treated aggressively. It usually occurs following the treatment of malignancies, but it has also been noted to occur spontaneously. Spontaneous tumor lysis syndrome (STLS) has been well described in hematologic malignancies [2]. However, its incidence in the setting of solid tumors is unknown, but is thought to be extremely rare. To date, less than 30 cases of STLS in solid tumors have been reported in the literature. To our knowledge, our case is only the third published case of STLS in a patient with small-cell lung cancer (SCLC).

## Case Report

We report the case of a 65-year-old man who presented to our hospital with complaints of generalized fatigue, anorexia, worsening abdominal distension and right upper quadrant pain, of 5 weeks duration. The patient described a daily abdominal pain, non-radiating, stabbing and intermittent in nature, worsening over the last week. He denied any associated fever, chills, night sweats, or recent infections. Prior medical history included hypertension and compensated alcoholic cirrhosis. Patient had a 25 pack-year smoking history, and had quit smoking 4 years ago. Family history was unremarkable.

On admission, the patient’s temperature was 96.6 °F, blood pressure was 74/47 mm Hg, and heart rate was 90/min. Physical examination revealed an ill-appearing man, in mild distress. Bilateral enlarged supraclavicular lymph nodes were noted. Abdominal exam was remarkable for marked hepatomegaly, with the liver extending more than 10 cm below the right costal margin. Spleen was not palpable. The rest of his examination was normal.

Initial laboratory analysis revealed a blood urea nitrogen (BUN) of 68 mg/dL and a creatinine of 3.92 mg/dL (the patient had a normal creatinine level at baseline). On further questioning, the patient denied decreased urine output, urinary symptoms, or over-the-counter, illicit or herbal medications use. Additional abnormal values included: potassium 7.4 mEq/L, carbon dioxide 12 mEq/L, uric acid 16.5 mg/dL, lactate dehydrogenase (LDH) 913 IU/L, inorganic phosphorus (IP) 4.3 mg/dL, and lactic acid 8.5 mol/L. The pertinent laboratory tests from admission are displayed in Table 1.

**Table 1:** Laboratory Values on Admission

Description	Results	Units	Reference range
Leukocyte count	12.23	× 10^9^/L	4.8 - 10.8
Hemoglobin	11.8	g/dL	14 - 18
Hematocrit	33.8	%	42 - 52
Platelets	405	× 10^9^/L	130 - 400
Sodium	131	mEq/L	135 - 146
Potassium	7.4	mEq/L	3.5 - 5
Chloride	101	mEq/L	98 - 110
Carbon dioxide	12	mEq/L	17-32
Anion gap	18	mEq/L	7-14
Glucose	136	mg/dL	70 - 110
Urea	66	mg/dL	10 - 20
Creatinine	3.92	mg/dL	0.7 - 1.5
Calcium	8.2	mg/dL	8.5 - 10.1
Inorganic phosphorus	4.3	mg/dL	2.1 - 4.9
Magnesium	2.3	mg/dL	1.8 - 2.4
Albumin	2.9	g/dL	3.0 - 5.5
Total bilirubin	5.9	mg/dL	0.2 - 1.2
Alanine aminotransferase	86	IU/L	0 - 45
Aspartate aminotransferase	92	IU/L	0 - 41
Alkaline phosphatase	498	IU/L	30 - 115
LDH	913	IU/L	60 - 200
Uric acid	16.5	mg/dL	4.8 - 8.7
Lactic acid	8.5	mmol/L	0.5 - 2.2

**Figure 1:**
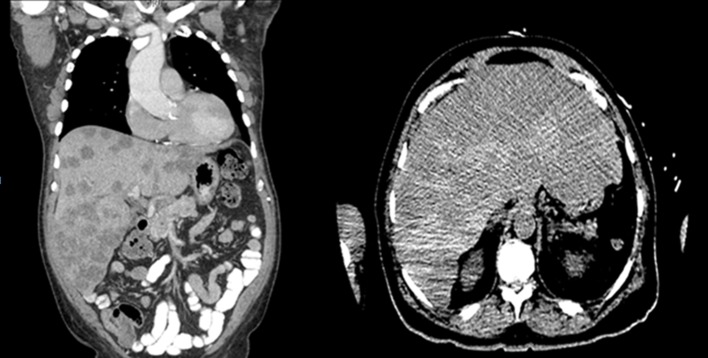
Innumerable hepatic masses.

**Figure 2:**
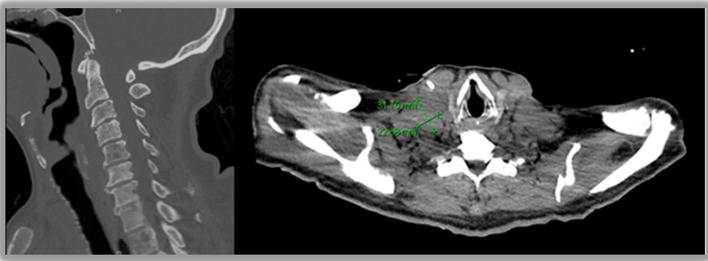
Metastases to cervical spine and supraclavicular lymph nodes.

A chest radiograph was unremarkable. Abdominal sonogram disclosed hepatomegaly with innumerable hepatic masses, and no hydronephrosis. A non-contrast computerized tomography (CT) scan of the chest, abdomen and pelvis confirmed the liver findings (Fig. 1), but also showed multilevel vertebral body metastases, as well as extensive bilateral supraclavicular, mediastinal, hilar and retroperitoneal adenopathy (Fig. 2). These findings were suggestive of either lymphoma or metastatic solid tumor.

The constellation of acute kidney injury (AKI), hyperkalemia, hyperuricemia, and high-normal IP level, led to a diagnosis of TLS. The patient was started on aggressive intravenous hydration and initially required vasopressor support for persistent hypotension. Blood pressure and renal function improved with continuous hydration, and patient was eventually weaned off vasopressors. Insulin and potassium binders were also administered for the hyperkalemia, and sodium bicarbonate was given for the metabolic acidosis. One dose of rasburicase was given for the severe hyperuricemia and allopurinol was added. Renal function, uric acid, and electrolyte disturbances all corrected over the next 7 - 10 days.

A peripheral blood flow cytometry was negative for lymphoma or any hematological malignancy. A supraclavicular lymph node biopsy revealed small cell carcinoma. On immunohistochemistry, the tumor was positive for pancytokeratin, CD56, synaptophysin, TTF1, and CK 20, and focally positive for chromogranin and napsin, supporting a primary lung malignancy.

On hospital day 9, patient was started on the first cycle of chemotherapy, with a combination of etoposide and carboplatin. He tolerated his first cycle of chemotherapy, was discharged and was scheduled to receive his next course of chemotherapy in 2 weeks.

Two weeks later, a repeat blood work showed a uric acid level of 4 mg/dL, a creatinine of 0.74 mg/dL, along with normal potassium and IP levels. A CT scan of chest, abdomen and pelvis done 5 weeks later showed significant improvement of mediastinal adenopathy, as well as an interval decrease in size of hepatic metastases. To date, the patient has received five out of six planned cycles of chemotherapy.

## Discussion

TLS is a catastrophic condition characterized by severe metabolic derangements in potassium, phosphorus, calcium and uric acid, leading to acute kidney failure, arrhythmias, central nervous system toxicity, and even death [2, 3]. It is precipitated by the destruction of tumor cells secondary to treatment with chemotherapy, with release of intracellular contents [3]. TLS has a predilection for hematologic malignancies such as Burkitt’s lymphoma, acute lymphocytic leukemia and diffuse large B-cell lymphoma, due to their sensitivity to therapy and their rapidly progressing nature [4-6]. However, cases of STLS have been described in malignancies not previously treated with chemotherapeutic agents. This phenomenon is thought to be due to rapid tumor necrosis causing the release of intracellular contents [3]. Like TLS, STLS is more commonly described in hematologic malignancies. However, recently, cases of STLS in solid tumors have been described in the literature, even though expert panels have designated solid tumors as low risk for the development of STLS [7, 8].

Although classified as low risk, a variety of solid tumors have been associated with TLS, including colorectal cancer, small-cell lung carcinoma, breast cancer, germ cell tumor, soft tissue sarcomas, and ovarian carcinoma. Even the very rare STLS has been described in solid tumors. An extensive literature review for STLS in non-hematologic solid tumors yielded 27 cases, including our case (Table 2) [9-31]. To the best of our knowledge, only two cases of STLS have been described in patients with SCLC, our case being the third [9, 10]. In the previously reported cases, both patients were found to have a significant tumor burden and expired shortly after diagnosis. Our patient, despite the large tumor burden, was aggressively and successfully treated.

**Table 2:** Cases of STLS in Solid Tumors [9-31]

Primary site and type of cancer	Tumor burden	Treatment	Outcome of TLS treatment	Year of publication
Adenocarcinoma of unknown primary [14]	- Liver and bone metastases - Bulky lymph nodes	- Hydration - Allopurinol - Alkalization	Death	1977
Inflammatory breast cancer [15]	- Large mass - Metastases to liver, bone, lungs, bone marrow	- Chemotherapy - Allopurinol	Survival	1995
Lung Adenocarcinoma [16]	- Large mass - Liver metastases	- Hydration - Allopurinol - Potassium and phosphate binders	Death	2000
Gastric Adenocarcinoma [17]	- Large mass > 7 cm in diameter - Liver metastases - Multiple lymph nodes	- Hydration - Chemotherapy - Allopurinol - Alkalization - Hemodialysis	Death	2001
Testicular germ cell tumor [18]	Case 1: - Liver and lung metastases - Bulky lymph nodes Case 2: - 20 × 25 cm retroperitoneal mass	- Surgery - Chemotherapy - Hemodialysis	Survival	2001
Colon cancer [11]	Liver metastases with necrosis	- Hydration - Allopurinol - Alkalization	Survival	2003
Hepatocellular carcinoma [11]	Large liver lesion	- Hydration - Allopurinol - Alkalization	Death	2003
Pheochromocytoma [11]	Large mass (20 cm diameter) with central necrosis	- Hydration - Allopurinol - Alkalization	Survival	2003
Prostate Cancer [19]	Liver and bone metastases	- Hydration - Allopurinol - Hemodialysis	Death	2007
Squamous cell Lung Cancer [12]	Advanced stage (stage IV)	- Hydration - Hemodialysis - Allopurinol - Chemotherapy	Survival	2009
Maxillary sinus squamous cell carcinoma [20]	Liver metastases	- Hydration - Rasburicase - Allopurinol	Death	2009
Germ cell tumor [21]	- 14 cm Retroperitoneal mass - Liver and lung metastases	- Hydration - Rasburicase	Death	2010
Germ cell tumor [22]	20 × 17 × 13 cm pelvic mass	- Hydration - Rasburicase - Chemotherapy - Surgery	Survival	2011
Small-cell lung cancer [10]	- Poorly differentiated tumor - Liver metastases	None	Death	2011
Melanoma [23]	Extensive metastases (including liver and spleen)	- Hydration - Hemodialysis	Death	2011
Hepatocellular carcinoma [24]	19.2 × 11 × 8 cm liver mass with extensive necrosis	- Hydration - Allopurinol - Hemodialysis	Death	2012
Small-cell lung Cancer [9]	- 4 cm obstructive lung mass - Multiple liver metastases and extensive lymph nodes	Hydration	Death	2012
Adenocarcinoma of unknown primary [25]	- Liver metastases, possible lung and bone metastases - Bulky retroperitoneal mass 14 × 13 cm	- Hydration - Rasburicase - Hemodialysis	Death	2012
Melanoma [26]	Liver metastases	Hydration	Death	2013
Renal cell carcinoma [13]	- 10 × 6.5 × 6.2 cm renal mass with necrosis - Liver, bone, pleura, bronchus, adrenal glands metastases	- Hydration - Allopurinol - Hemodialysis	Death	2014
Cholangiocarcinoma [27]	Liver metastases	- Hydration - Allopurinol	Death	2014
Hepatocellular carcinoma [28]	- 14 × 14 cm liver mass -Multiple extensive lymph nodes	- Hydration - Alkalization	Unknown (hospice/palliative care)	2014
Gastric Adenocarcinoma [29]	- Liver, bone and adrenal gland metastases - Multiple lymph nodes	- Hydration - Allopurinol - Hemodialysis	Survival	2014
Skin Adenocarcinoma [30]	- Multiple subcutaneous, liver, kidney and adrenal gland metastasis	- Allopurinol - Potassium binders - Alkalinization	Death	2014
Pancreatic Adenocarcinoma [31]	- 6.7 × 10 cm mass - diffuse liver metastasis and extensive lymph nodes	- Hydration - Allopurinol - Rasburicase	Death	2015
Small-cell lung cancer (our case)	- Liver, bone metastasis - Extensive lymph nodes	- Hydration - Rasburicase - Allopurinol - Alkalization - Potassium binders - Chemotherapy	Survival	2015

The most widely used diagnostic criterion for TLS is the Cairo-Bishop criterion [3]. According to this classification, to fulfill the diagnosis, at least two laboratory criteria must be present for 3 days before treatment or up to 7 days after treatment (Table 3). Clinical TLS is defined as the presence of at least one clinical finding not explained by treatment with chemotherapy or its side effects, such as cardiac arrhythmia and seizures (Table 4). The Cairo-Bishop criterion has important limitations as it is restricted to treatment with chemotherapy, and excludes TLS caused by radiation therapy or tumor embolization. Our patient had evidence of laboratory TLS as he had elevated uric acid and potassium levels. The superimposed AKI also led to a diagnosis of clinical TLS.

**Table 3:** Cairo-Bishop Laboratory Criterion

Variable	Value	Change from baseline
Uric acid	≥ 8 mg/dL	25% increase
Phosphorus	≥ 6 mEq/L	25% increase
Potassium	≥ 4.5 mg/dL for adults and 2.1 mmol/L	25% increase
Calcium	≤ 7 mg/dL	25% decrease

Diagnosis established by the presence ≥ 2 laboratory abnormalities 3 days before chemotherapy or 7 days after chemotherapy, if the patient has or will receive hydration and uric acid lowering agents.

**Table 4:** Cairo-Bishop Clinical Criterion

Variable	Grade 0	Grade 1	Grade 2	Grade 3	Grade 4	Grade 5
Creatinine	None	1.5 times ULN	> 1.5 - 3.0 times ULN	> 3.0 - 6.0 times ULN	> 6.0 times ULN	Death
Cardiac arrhythmia	None	No intervention	Non-urgent medical intervention indicated	Symptomatic and incompletely controlled medically or device-controlled	Life-threatening (arrhythmia with HF, shock, etc.)	Death
Seizure	None	None	One generalized seizure, seizures controlled by anticonvulsants or infrequent focal motor seizures not interfering with ADL	Seizures in which consciousness is altered; poorly controlled seizure disorder, with breakthrough generalized seizures despite medical intervention	Seizure which is prolonged, repetitive or difficult to control	Death

Diagnosis established by the presence of at least one clinical finding not explained by treatment with chemotherapy or its side effects.

In TLS and STLS, the sudden release of intracellular contents causes a myriad of serious and potentially life-threatening sequelae. Increased serum potassium leads to skeletal muscle dysfunction and arrhythmias including ventricular tachycadia [32]. Released uric acid causes renal endothelial dysfunction and inflammation due to smooth muscle cells exposed to uric acid, releasing pro-inflammatory cytokines [33]. There is also evidence of local ischemia due to uric acid scavenging nitric oxide, causing vasoconstriction and impairment of local renal repair mechanisms [33, 34]. Most significantly, uric acid precipitates within the urinary tract causing renal dysfunction and decreased excretion of phosphate [35]. The excess phosphate binds to calcium forming calcium phosphate which also accumulates within the renal tract further exacerbating renal impairment and metabolic acidosis as well as leading to hypocalcemia [11, 35]. The decrease in free serum calcium causes seizures, tetany, psychiatric complaints and arrhythmias [32]. It has been observed that patients with STLS have less hyperphosphatemia than patients with induced TLS due to increased phosphate uptake by the rapidly dividing cells of the tumor [11, 32, 36].

Although the rapid release of electrolytes from lysing tumor cells can have devastating effects, homeostatic mechanisms can often compensate if kidney function is intact [35]. Thus, the development of AKI is a strong predictor of mortality. The 6-month mortality for patients with TLS and AKI is 66% compared to 21% (P = 0.0006) in those patients with no evidence of AKI [37]. Other potential risk factors for STLS in solid tumors include increased tumor burden, evidence of large-sized masses and lymph node involvement, pre-existing hyperuricemia, elevated LDH, metastatic disease (especially liver and bone metastases), and external compression of the urinary tract leading to azotemia [5, 37-40] (Table 5).

**Table 5:** Risk Factors for the Development of TLS and STLS

Extensive tumor burden (bulky disease or disseminated disease)
Rapidly proliferating tumor
Extensive bone marrow involvement
Hepatic metastases
Highly chemosensitive malignancy
Elevated LDH level
Elevated uric acid level
Impaired renal function
Exposure to nephrotoxic drugs or uric acid excretion inhibiting drugs
Extrinsic compression of the urinary tract by the tumor
Dehydration, Infection or urinary obstruction

Given the aforementioned risk factors, prevention of AKI is essential, therefore volume expansion with oral or intravenous fluids is paramount [12, 13, 37]. It is also prudent to avoid nephrotoxins such as non-steroidal anti-inflammatory drugs, iodinated contrast and vasoconstrictive medications [32]. Patients with elevated uric acid and LDH levels at baseline can also be treated with a xanthine oxidase inhibitor, such as allopurinol, and a urate oxidase, such as rasburicase [12]. It is hypothesized that in the setting of solid tumors with extensive metastases, LDH and uric acid levels may be used to determine patients with asymptomatic STLS [12]. Accurate and timely recognition of risk factors allows for identification of high-risk patients and initiation of prophylactic measures to prevent STLS.

Once STLS develops, the focus of treatment is to reestablish normal concentration of extravasated solutes. The cornerstone of therapy is volume expansion to increase kidney excretion of solutes and decrease the risk of crystal formation. Hyperkalemia can be treated with intravenous calcium, intravenous insulin, inhalation of beta-2-agonists, intravenous sodium bicarbonate and sodium polystyrene [41]. Management of hyperphosphatemia involves restriction of phosphorus intake and the use of phosphate binders [42]. STLS is also characterized by hypocalcemia; however, it should not be treated with calcium supplementation due to calcium phosphate crystallization within the kidneys, but should be administered in the case of arrhythmia, cardiac arrest and seizure [42]. For the treatment of hyperuricemia, alkalinization of urine had been proposed, as the solubility of uric acid is greater at a neutral pH as opposed to the acidic pH of urine [32]. However, this treatment modality is nowadays controversial. Allopurinol prevents the generation of uric acid and maintains normal uric acid levels [43, 44]. More recently treatment with rasburicase, which converts urate into water soluble allantoin, has been proven to be effective [32, 43]. Numerous studies have demonstrated significant reductions in uric acid level even compared to allopurinol [45]. Lastly, though hopefully avoidable, in patients with severe AKI and STLS, there may arise a need for hemodialysis. Early or urgent hemodialysis is indicated for life-threatening hyperkalemia and severe hyperphosphatemia. It is estimated that one-third of patients with clinical TLS will require hemodialysis [46].

However, despite therapy, 15% of diagnosed cases of TLS are fatal [46]. These numbers are greater in those patients in whom diagnosis is missed or delayed, therefore a high index of suspicion has be maintained in patients with any kind of malignancy with derangements in kidney function, uric acid and electrolytes.

### Conclusion

TLS is an oncologic emergency that can arise as a result of cancer therapy, or spontaneously. It is most commonly associated with hematologic malignancies and is a rare phenomenon in solid tumors, especially SCLC. In patients with non-hematological malignancies and risk factors, including renal impairment and large tumor burden, close monitoring of clinical status and laboratory values is essential, not only to diagnose and treat STLS, but also to prevent this catastrophic condition.
